# Research trends and hotspots on septic shock: a bibliometric study and visualization analysis

**DOI:** 10.3389/fmed.2024.1490462

**Published:** 2024-11-27

**Authors:** Sitong Wang, Nan Li, Ben Ma, Shuang Zhu, Yu Zhou, Ruihang Ma

**Affiliations:** Department of Emergency Medicine, General Hospital of Northern Theater Command, Shenyang, Liaoning, China

**Keywords:** septic shock, bibliometrics, CiteSpace, VOSviewer, Web of Science

## Abstract

**Background:**

Septic shock, the most severe stage of sepsis, causes potential circulatory failure and abnormal cell metabolism which are severe enough to affect prognosis, increase mortality, and impose significant burdens on the medical system. Despite a growing number of studies exploring the pathophysiology, epidemiology, and risk factors, research trends and hotspots in septic shock remain lacking. This study aims to create a visual knowledge map, identify research hotspots, and predict prospective trends based on bibliometric analysis.

**Methods:**

We searched for publications related to septic shock in Web of Science Core Collection up to June 15, 2023. CiteSpace5.5 R2, VOS viewer and Pathfinder were used to evaluate the annual publications, countries, institutions, journals and keywords. We also analyzed the collaboration among countries, institutions and authors, and identified research hotspots and frontiers.

**Results:**

A total of 4,208 English papers were included in the analysis, and the annual publication displayed a slow upward trend. In terms of publication volume, the top three countries were the United States, France, and Germany, and the University of Pittsburgh (the United States) ranked first (*n* = 85) among all institutions, with Jeanlouis Vincent from Erasmus Medical Center (Netherlands) as the most published author (*n* = 32). According to the collaborative network, the United States had the highest level of cooperation, and the University of Pittsburgh, the University of Toronto, and Columbia University were the institutions with the most foreign cooperation. Additionally, the co-author network revealed that scholars such as Jeanlouis Vincent, Rinaldo Bellomo, and Djillali Annane, had the strongest collaborations. The co-citation network showed that the top 3 most cited articles were: Singer M (2016), Rhodes A (2017), Dellinger RP (2013), and the top 3 most cited journals were *Crit Care Med* (3,664 times), *N Engl J Med* (3,207 times), *Intens Care Med* (3,096 times) in this field. In the keyword co-occurrence network, the most frequent keywords were “septic shock” (2531), “sepsis” (1667), and “mortality” (569), indicating the current research hotspots. Pathobiology, fluid therapy, and endotoxic septic shock were emerging trends in research.

**Conclusion:**

By using bibliometrics, this study reviewed the studies in septic shock and revealed the hotspots and cutting-edge trends, including the pathogenesis of complications, the development of new biomarkers, the timing and methods of alternative treatments, and the rehabilitation trajectory, etc., which provided a reference for subsequent studies in septic shock.

## 1 Introduction

Sepsis is a life-threatening multi-organ dysfunction caused by a dysregulated host response to infection and is one of the leading causes of death in critically ill patients worldwide (1). Without prompt treatment, sepsis can rapidly progress into septic shock in a short period of time. Septic shock refers to an acute circulatory failure characterized by severe hypotension and hyperlactatemia requiring adequate vasoactive drug maintenance and fluid resuscitation (2). The potential circulatory failure and cellular metabolic abnormalities caused by septic shock contributed to increased mortality (3). Scholars unanimously agree that septic shock is a critical disease associated with a greater risk of mortality than with sepsis alone (4). Therefore, how to prevent and treat septic shock has become an important theme in critical care and emergency medicine. Although the “Guidelines for Management of Sepsis and Septic Shock” (5) clearly state that the principles of initial management are to provide cardiorespiratory resuscitation and mitigate the threats of uncontrolled infection, the components required for optimizing resuscitation, such as the fluids selection, the hemodynamic monitoring, and adjunctive vasoactive drugs, are still hot topics in the current research field and clinical trials. The emerging interest in septic shock prompted our study to investigate in current status, research trends and related hotspots from a macro view.

Bibliometrics (6) is a quantitative assessment that uses mathematical and statistical methods to evaluate the contribution of scientific literature. It is also an important way to reveal research trends and predict research hotspots in a certain special field of study. Bibliometric analysis of post-stroke dysphagia rehabilitation, ophthalmology, and the post-intensive care syndrome (PICS) by researchers have offered insightful study topics of interest (7, 8). In recent years, septic shock has received extensive attention, while the research hotspots, patterns and trends have not yet been clarified. Therefore, we conducted a bibliometric analysis of the literature on septic shock in the Web of Science Core Collection (WoSCC) database over the past 27 years. We summarized the countries, institutions, journals, authors, and keywords, aiming to understand the most influential ones in this field, obtain highly cited articles and keywords, form clustered themes, analyze current research hotspots, and reveal the direction of development frontiers. This study not only provides readers and researchers with an overall visual knowledge map and significant insights into the topic of septic shock rehabilitation, but provides meaningful references for future investigation.

## 2 Materials and methods

### 2.1 Data source and search strategy

**Figure 1:**
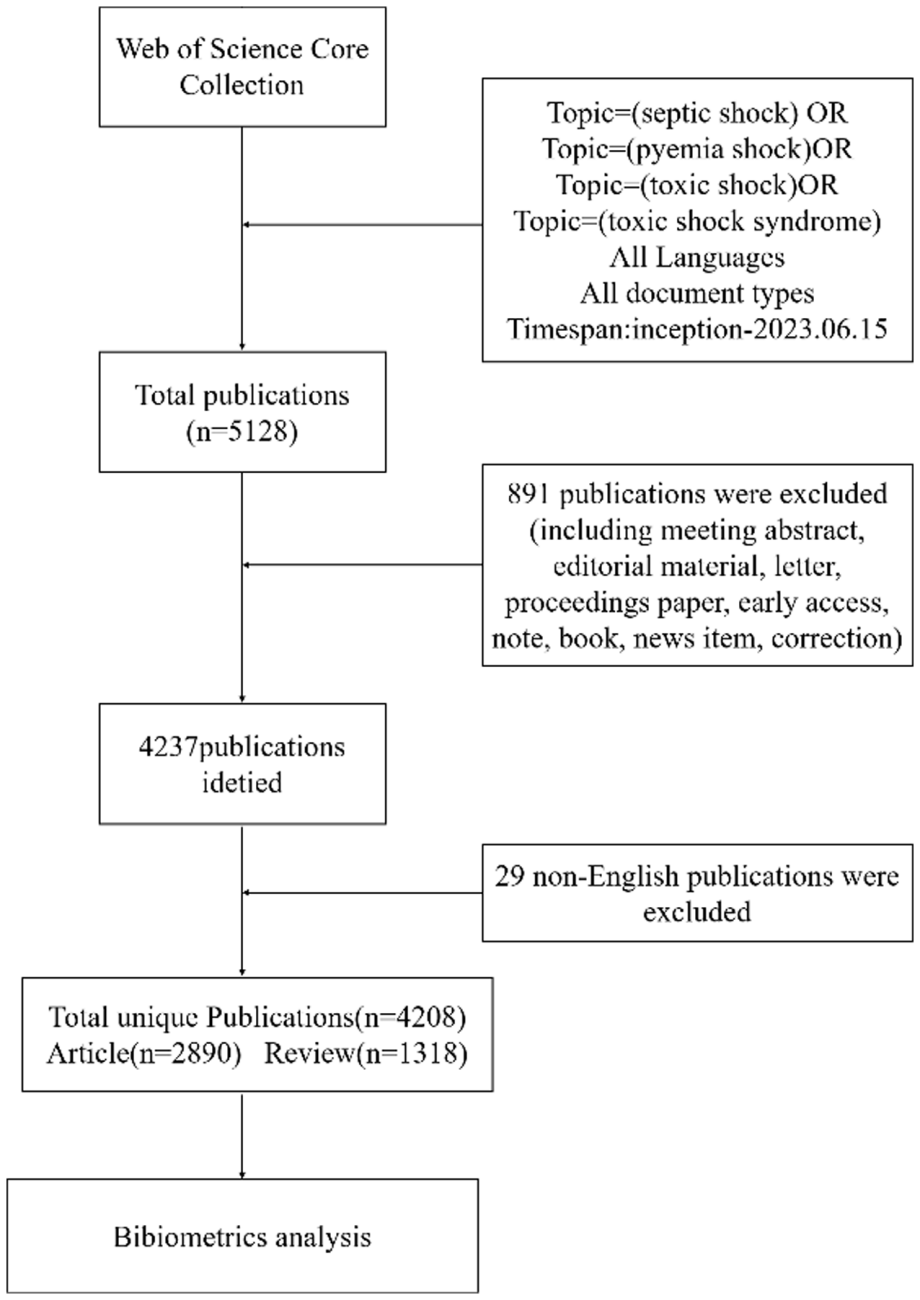
The flow chart of searching papers in databases.

To collect relevant data, we searched the WoSCC from its inception until June 15, 2023. The search strategy was as follows: “septic shock” OR “pyemia shock” OR “toxic shock” OR “toxic shock syndrome.” The search content included title, abstract, author and keywords and keywords plus. Only original articles and reviews were included and the language was limited to English. A total of 5,128 documents related to septic shock were obtained during the initial inspection, and information in “Core Collection Full Record Details” section were downloaded. Retrieved data were imported into Endnote X9 software for deduplication. After excluding the literature irrelevant to the research topic, duplications, and literature for which the full text was unavailable, a total of 4,208 publications (2,890 articles and 1,318 reviews) met the inclusion criteria. The flow chart of the literature screening is shown in Figure 1.

### 2.2 Bibliometric analysis and visualization

After the retrieval data was deduplicated by Endnote X9, CiteSpace5.5 R2 and VOS viewer were used for analysis, quantification, and visualization. In the parameter settings of CiteSpace5.5 R2, the time span was set from January 1996 to December 2023, with 1-year time slices and a threshold of top 10%. The main contents of the analysis included countries, journals, authors, institutions, cited publications, and keywords. Next, the institutions, authors, co-citation publications were presented in a network weighted by co-occurrence to further construct a knowledge map of septic shock quantitatively and qualitatively. The networks were pruned to a Pathfinder network scaling, and keyword co-occurrence analysis with the assistance of the VOS viewer.

## 3 Results

### 3.1 Number of publications and international cooperation

**Figure 2:**
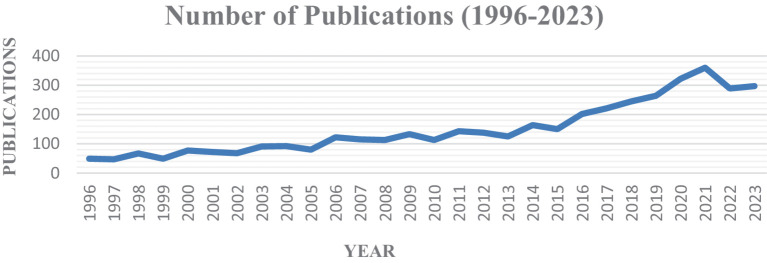
Annual trend in publications of septic shock.

Our investigation included 4,208 publications from the inception of the WoSCC database to the date of retrieval. The earliest publication in this field was an article entitled Cardiac Dysfunction During Septic Shock by Parrillo JE (9) in *Clinics in Chest Medicine* in 1966. In the early days (1996–2015), the volume of papers on septic shock increases slowly, but there has been a notable upsurge since 2016, indicating that this area has become a hotbed of research (Figure 2).

**Figure 3:**
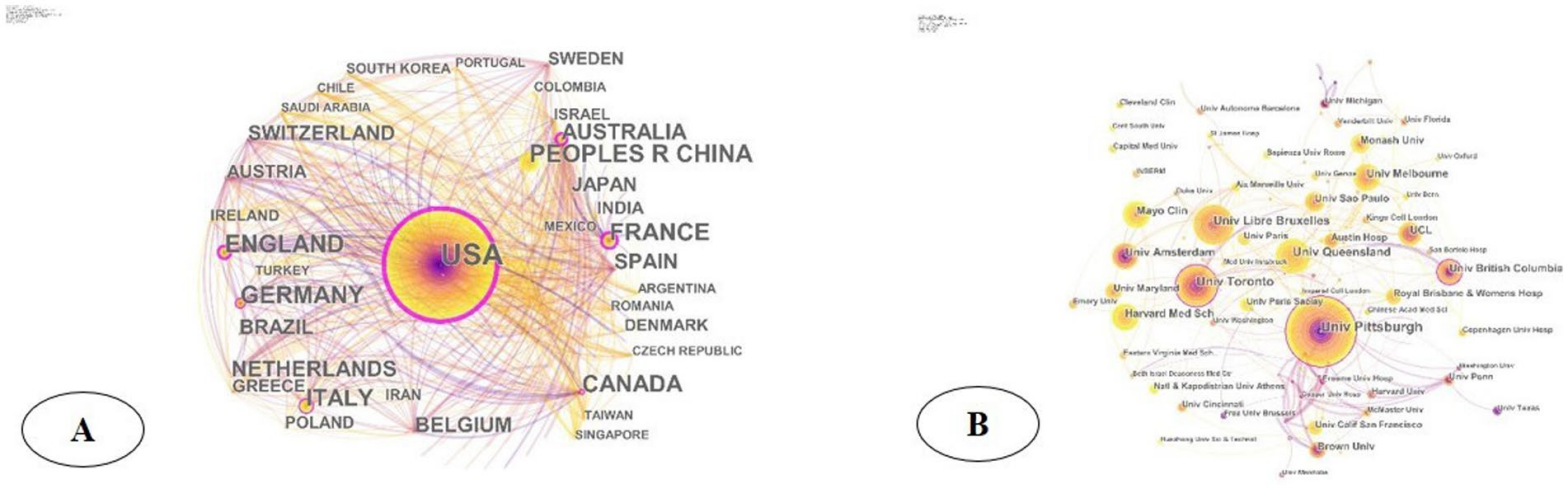
(A) Network of international cooperation of septic shock in geographic visualization; (B) institutional cooperation network diagram of septic shock.

In total, 2,896 institutions in 261 countries/regions conducted research related to septic shock, and the top 10 contributing countries and institutions were shown in Table 1. The United States had the largest number of papers published in this field (*n* = 1,371), followed by France (*n* = 341), Germany (*n* = 305), the United Kingdom (*n* = 304), and China (*n* = 299). The top 10 countries published 3,681 papers, accounting for 87.5% of the total volume. In the national cooperation network, the United States occupied a dominant position in research and led the trend. As shown in Figure 3A, the United States was central to the international cooperation network and connected closely with other countries/regions (France and Australia as its main collaborators). Although Germany and China ranked in the top 3 and top 5, respectively, both had few collaborations with other countries. As a contrast, the United Kingdom (4th) and Italy (6th) had more extensive foreign collaborations.

**Table 1:** Top 10 most productive countries and institutions on septic shock.

Rank	Country/region	H-index	Count (%)	Institution	H-index	Count (%)
1	USA	44	1,371 (32.6%)	Univ Pittsburgh	12	85 (2.02%)
2	France	26	341 (8.10%)	Univ Toronto	12	50 (1.17%)
3	Germany	15	305 (7.25%)	University of Bruxelles	8	49 (1.16%)
4	U.K.	21	304 (7.22%)	Univ Queensland	2	43 (1.02%)
5	China	7	299 (7.11%)	Univ Amsterdam	1	34 (0.81%)
6	Italy	12	281 (6.68%)	Mayo Clin	1	33 (0.78%)
7	Canada	13	247 (5.87%)	Harvard Med Sch	3	32 (0.76%)
8	Australia	28	196 (4.66%)	University of Melbourne	2	31 (0.74%)
9	Netherlands	7	190 (4.52%)	University College London	1	29 (0.69%)
10	Belgium	0.06	147 (3.50%)	Univ British Columbia	11	29 (0.69%)

The research institutions with the largest number of publications were the University of Pittsburgh (*n* = 85), the University of Toronto (*n* = 50), the Free University of Brussels (*n* = 49), and the University of Queensland (*n* = 43) (Table 1). The cooperation network of institution showed (Figure 3B) that institutions such as the University of Pittsburgh, the University of Toronto, Columbia University, the University of Amsterdam, the Free University of Brussels, the University of Queensland, and the Mayo Clinic had more external cooperation and were more cohesive than other institutions in the field of septic shock research. Among them, the nodes representing the University of Pittsburgh, the University of Toronto, and the Columbia University Center have purple outer circles, indicating their higher centrality in the cooperation network and extensive cooperation.

### 3.2 Journal and field distribution

**Figure 4:**
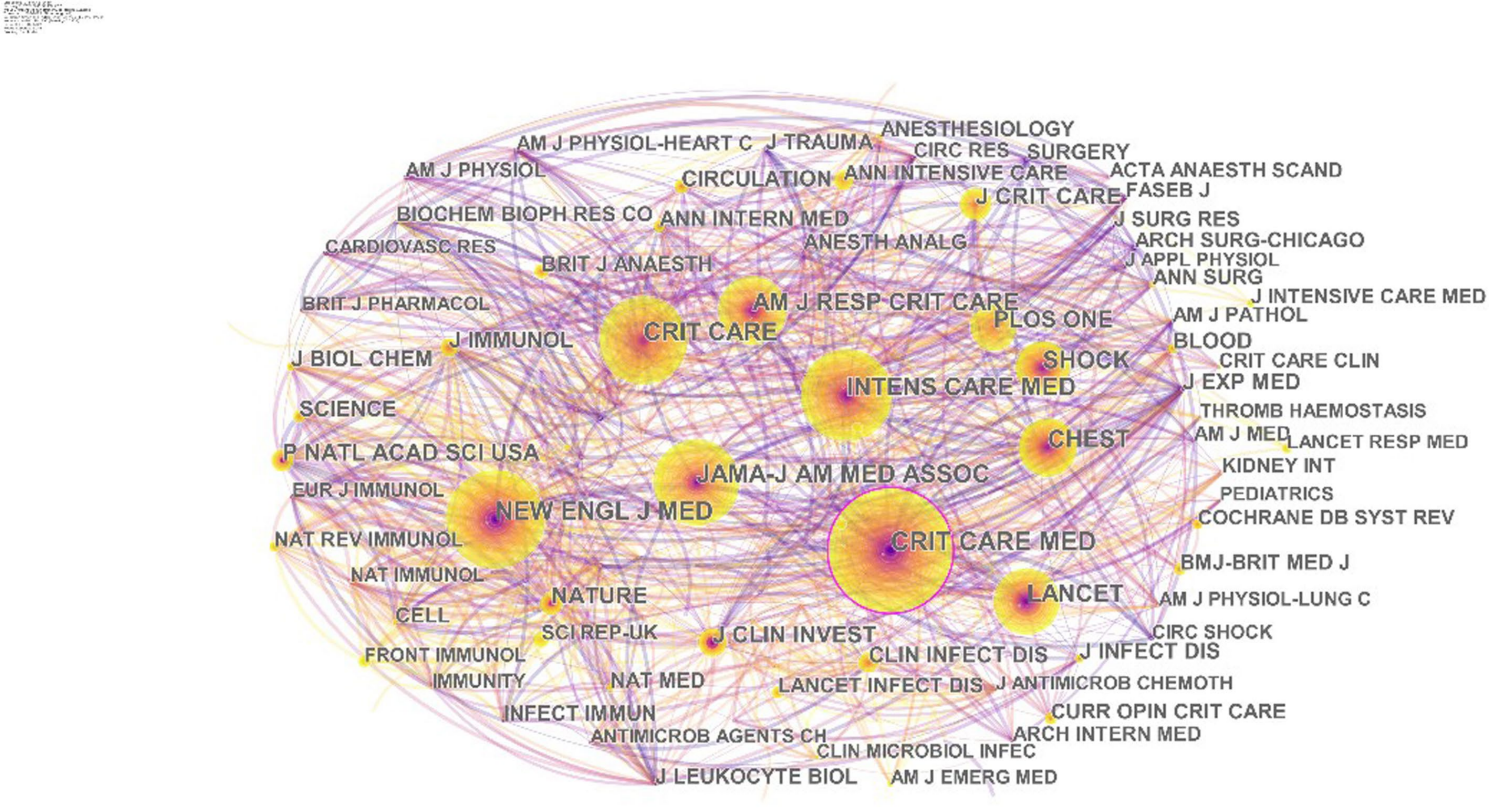
Co-citation analysis of journals in the field of septic shock.

Since 1998, papers with the theme of septic shock have been published in 382 academic journals. The most cited articles in a field are often considered the most impactful findings that influence further research. The top 10 journals with the high co-citation frequency were *Crit Care Med* (frequency = 3,664), *N Engl J Med* (frequency = 3,207), *Intens Care Med* (frequency = 3,096), *JAMA-J AM MED ASSOC* (frequency = 2,972), *Crit Care* (frequency = 2,676), *Lancet* (frequency = 2,392), *AM J RESP CRIT CARE* (frequency = 2,253), *Chest* (frequency = 2,220), *Shock* (frequency = 1,988), and *J CLIN INVEST* (frequency = 1,471) (Figure 4). The distribution of research fields, as expected, was dominated by critical care medicine and emergency medicine. According to journal impact factor (JIF), journal citation reports (JCR) divide journals of the same subject category into four equal parts, with the top 25% of journals classified as Q1, 25–50% of journals classified as Q2, and so forth (10). More than half of the top 10 citing journals were classified in Q1, including some of the world-famous medical journals such as *N Engl J Med* and *Lancet*.

### 3.3 Authors and author co-citation analysis

**Figure 5:**
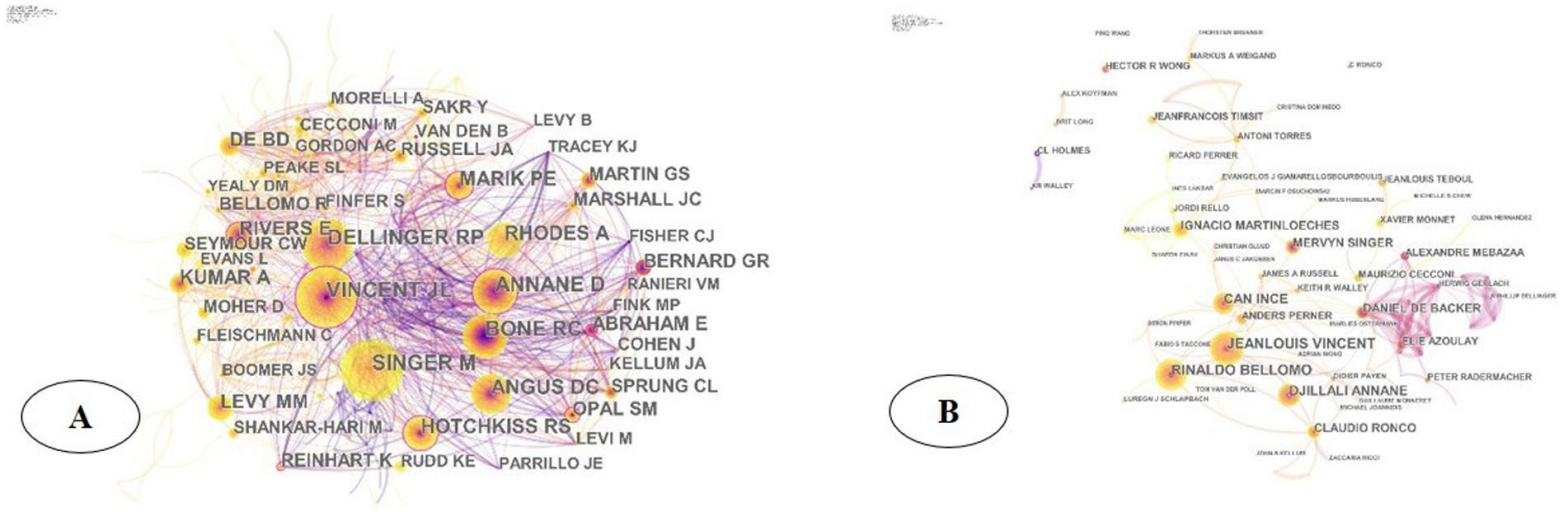
(A) Author Co-citation Network in the field of septic shock; (B) collaborative networks of co-author analysis in the field of septic shock.

To ensure the accuracy of data analysis, we initially used the Researcher ID and ORCID of WoS to identify each author. Both systems assign a unique identifier to each author, effectively resolving problems caused by name ambiguity. The scholar with the most publications was Jeanlouis Vincent of Erasmus Medical Center in the Netherlands (*n* = 32), followed by Rinaldo Bellomo (*n* = 32) and Djillali Annane (*n* = 32). According to the analysis, Professor Vincent JL of Erasmus Medisch Centrum in the Netherlands (frequency = 1,108) ranked first in citation frequency, and Society of Critical Care Medicine and the European Society of Intensive Care Medicine (frequency = 960) and Professor Annane D of the University of Rennes in France (frequency = 957) were the second and third most cited authors, respectively, indicating their influence and core position in this field. The highest centrality distribution of researchers, marked by the purple circles outside the nodes, were Vincent JL and Annane D (>0.1), indicating their influence and cohesion in septic shock (Figure 5A). In the collaborative network, Jeanlouis Vincent, Rinaldo Bellomo, Djillali Annane, etc., who had a higher frequency of collaboration, formed a closely related collaboration network, while Daniel DE Backer, Elie Azoulay, Alexandrei Mebazaa, etc. formed a loosely one (Figure 5B).

### 3.4 Co-citation analysis of literature

Literature co-citation analysis is one of the most attractive functions of CiteSpace, which is usually used to determine the focus in a specific field and reflects research hotspots and frontiers. The most cited literature was published by Singer M and many other scholars in *JAMA-J AM MED ASSOC* in 2016, entitled: The Third International Consensus Definitions for Sepsis and Septic Shock (Sepsis-3) (frequency = 907) (11). This was the third international consensus on sepsis and septic shock. This consensus re-examined the definitions and clinical standards of sepsis and septic shock in pathobiology (including organ morphology, cell biology, immunology, etc.), treatment and epidemiology since 2001. The new consensus aligned with clinical practice experience, providing consistency and standardization for epidemiology and clinical trials in this field. Sepsis-3 is helpful for early identification and management of patients with or at risk of sepsis/septic. The second most cited paper was Surviving Sepsis Campaign: International Guidelines for Management of Sepsis and Septic Shock: 2016 (frequency = 394) (12) published by Rhodes A, i.e., the International Guidelines for Sepsis and Septic Shock (2016). Based on the 2012 guideline, this revision (2016) convened 55 international experts from 25 international organizations, and updated the contents from five aspects: hemodynamics, infection, adjuvant therapy, fluid resuscitation and ventilation. It also generated 93 evidence summaries on the early management and resuscitation of patients with sepsis or septic shock, and reached substantial consensus, which laid an evidence-based foundation for the clinical manifestations, early intervention and prognosis. The third most cited publication was Dellinger RP (2013) (frequency = 268) (13) (Table 2).

**Table 2:** Top 10 most cited papers in the field of septic shock.

Rank	Count of citations (*n*)	Centrality	Highly cited articles
1	907	0.13	SINGER M, 2016, JAMA-J AM MED ASSOC, V315, P801, DOI 10.1001/JAMA.2016.0287 (11)
2	394	0.04	RHODES A, 2017, CRIT CARE MED, V45, P486, DOI 10.1097/CCM.0000000000002255 (12)
3	268	0.05	DELLINGER RP, 2013, INTENS CARE MED, V39, P165, DOI 10.1007/S00134-012-2769-8 (13)
4	206	0.25	BERNARD GR, 2001, NEW ENGL J MED, V344, P699, DOI 10.1056/NEJM200103083441001 (43)
5	181	0.01	FLEISCHMANN C, 2016, AM J RESP CRIT CARE, V193, P259, DOI 10.1164/RCCM.201504-0781OC (18)
6	180	0.06	RIVERS E, 2001, NEW ENGL J MED, V345, P1368, DOI 10.1056/NEJMOA010307 (44)
7	179	0.01	RUDD KE, 2020, LANCET, V395, P200, DOI 10.1016/S0140-6736(19)32989-7 (19)
8	172	0.18	ANNANE D, 2002, JAMA-J AM MED ASSOC, V288, P862, DOI 10.1001/JAMA.288.7.862 (38)
9	172	0.02	PEAKE SL, 2014, NEW ENGL J MED, V371, P1496, DOI 10.1056/NEJMOA1404380 (45)
10	158	0.05	ANGUS DC, 2001, CRIT CARE MED, V29, P1303, DOI 10.1097/00003246-200107000-00002 (23)

### 3.5 Keywords

#### 3.5.1 High-frequency keywords

**Figure 6:**
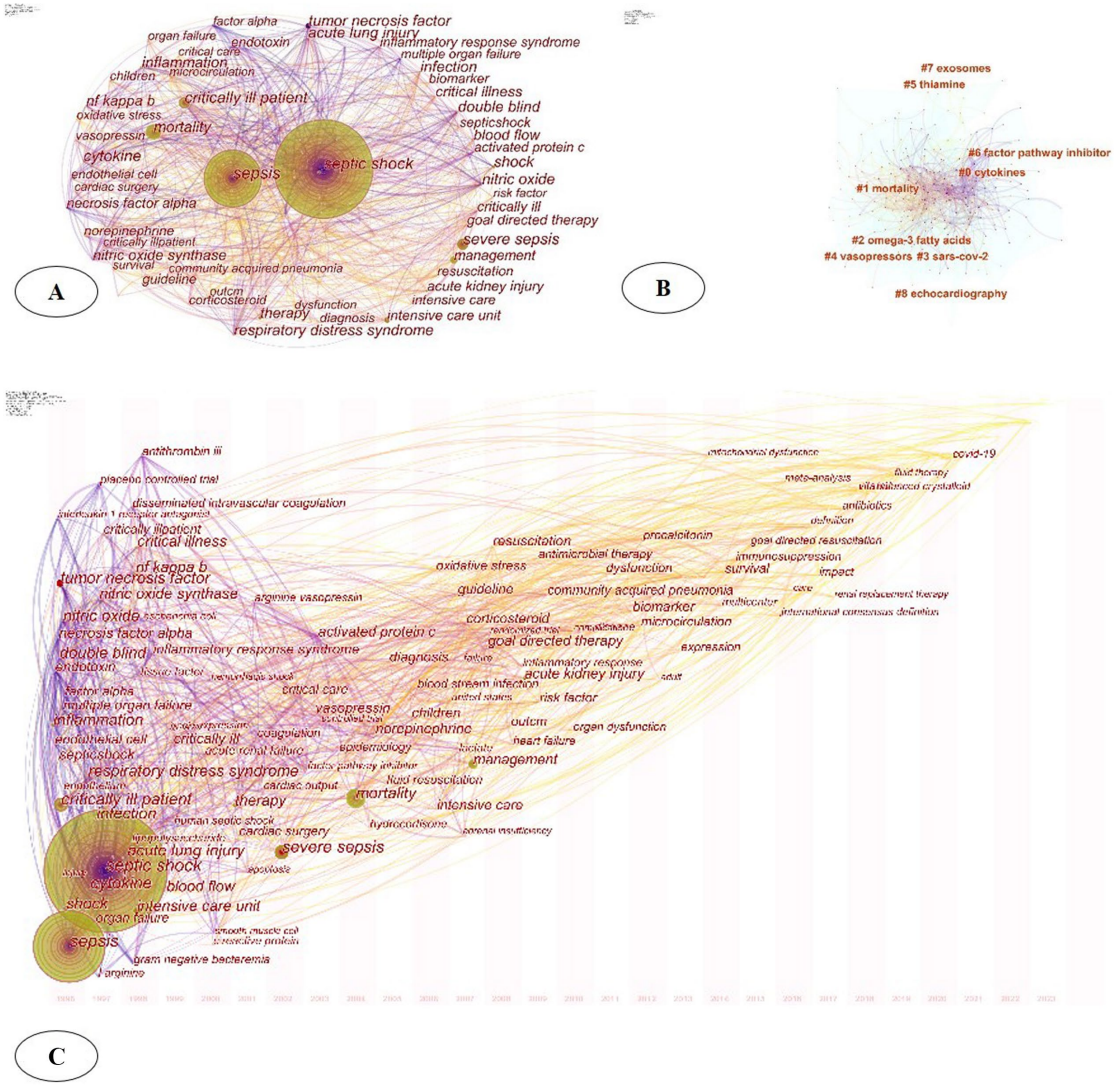
(A) Keywords analysis of septic shock; (B) keyword clustering analysis; (C) spatiotemporal evolution trend of keywords.

Keywords are natural languages that reflect the core content of the literature. High-frequency keywords indicate the hotspots and trends in the research field (14). According to frequency analysis, the top 20 keywords are shown in Table 2. The larger the node in the co-occurrence network, the higher the frequency of the keyword, and the more likely it was to be a hotspot. Figure 6A showed that the larger nodes were septic shock (frequency = 2,531), sepsis (frequency = 1,667), mortality (frequency = 569), critically ill patient (frequency = 539), and severe sepsis (frequency = 538). Among them, the septic shock and sepsis nodes had the largest annual rings, indicating the highest occurrence. Notably, the map did not present a single disease of sepsis or septic shock, but involved prognosis, risk factors, infection, tumors, renal injury, management, etc., suggesting that the multidisciplinary participation of septic shock.

#### 3.5.2 Keyword clustering

Cluster labels were extracted using keywords to obtain a cluster map. Through keyword clustering analysis the relationship between keywords was clarified (15). The modularity (Q how well the communities are separated) and the mean silhouette scores (Show appropriate each node is assigned to its cluster) are two important metrics that evaluate the overall structural properties of the network. In our study. *Q* = 0.385 (>0.3) means reasonably division of the clusters. *S* = 0.6852 suggested that the homogeneity of these clusters on average was ideal. The clustering effect parameters of this study were *S* = 0.6852 and *Q* = 0.385. From Figure 6B the main keyword clusters were divided into 9 clusters namely: #0 cytokines, #1 mortality, #2 omega-3 fatty acids, #3 scars-cov-2, #4 vasopressors, #5 thiamine, #6 factor pathway inhibitor, #7 exosomes, and #8 echocardiography

#### 3.5.3 Spatiotemporal evolution trend of keywords

The time zone chart displays the new keywords that appear each year, which provides a perspective on the temporal evolution of the research focus. Figure 6C shows that in the past 30 years, researchers have been committed to the pathogenesis, pathophysiology, epidemiology, risk assessment, complication prevention and treatment of septic shock (including acute respiratory distress syndrome, acute renal injury, liver injury, myocardial injury and coagulation disorders, etc.). After 2010, the shift in focus toward keywords such as “goal directed therapy” and “fluid resuscitation” appeared and had sustained interest. Since 2021, keywords such as “COVID-19” and “infectious diseases” appeared, representing the emerging research directions and cross-border multidisciplinary trends in the future.

#### 3.5.4 Keyword emergence

**Figure 7:**
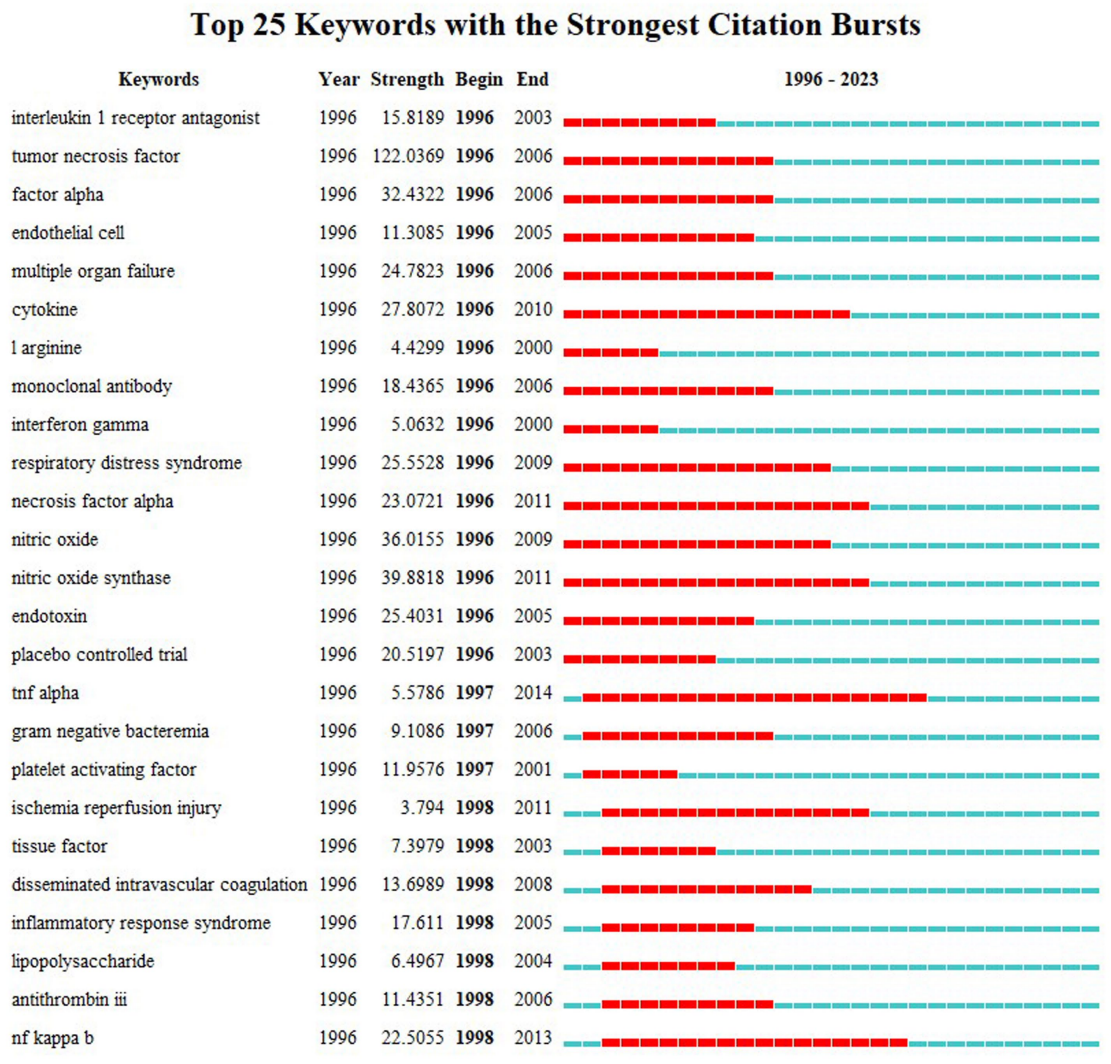
Top 25 keywords with the strongest citation bursts.

Keyword emergence analysis provides a keyword burst detection feature, which helps track the evolution of research hotspots over time and forecast future developments in the field (7). Figure 7 highlights the top 25 keywords with the strongest citation bursts of septic shock research in the past three decades. Among them, sustained hotspots such as cytokine, necrosis factor alpha, nitric oxide synthase, ischemia reperfusion injury, NF kappa b, disseminated intravascular coagulation, etc. had the longest emergence time, all of which involved internal mechanism and pathophysiology. From the initial “inflammatory response,” to the “compensatory anti-inflammatory response syndrome” (16), septic shock triggers a more complex, variable and persistent host response, obviously. The pro-and anti-inflammatory mechanisms help the infection clearance and tissue recovery, and on the other hand, aggravate organ damage and secondary infection. Therefore, the relationship between the patient’s specific response, the pathogenic pathogen (viral load and intensity) and the host (genetic characteristics and comorbidities) is still the hotspot of scholars in related fields.

## 4 Discussion

Septic shock, a shock caused by sepsis, is a common critical illness in the ICU (17). It is often associated with severe hypotension, microcirculatory disorders, organ perfusion insufficiency, and even multiple organ failure (heart, liver, kidney, lung, brain). Sepsis and septic shock claim millions of lives and constitute a huge global health burden around the world every year (18). Despite improved healthcare system and raised public awareness (19), sepsis remains a major threat to human health and is listed as a major global health concern by the World Health Organization (WHO). Since the medical burden leads to the substantial cost, sepsis and septic shock also impose a significant economic global burden on health care systems, patients and families. Reports found that approximately 48.9 million cases of sepsis were reported worldwide in 2017 alone, and 11 million people eventually died of this disease, accounting for 19.7% of all diseases (20). By 2021, there will be more than 18 million cases of septic shock globally (20, 21). The short-term mortality rate is as high as 60% in the case of septic shock (22). Given that the potential circulatory disorders and cell metabolic abnormalities significantly increase the mortality rate, how to prevent, diagnose and treat septic shock has become a hot topic in critical care medicine. To provide public health policy and clinical practice guidelines, we should gain a macro perspective of septic, including morbidity, mortality, research hotspots, cutting-edge trends, and clinical drug research. We aim to reduce the global burden through early detection, targeted therapies, and timely initiation of evidence-based treatment, which necessitates a comprehensive review of the literature on septic shock. Bibliometric analysis uses quantitative methods to depict research in a certain field, objectively evaluates the research hotspots, and reveals directions that have not yet been studied (19) Based on the software such as CiteSpace5.5 R2 and VOS viewer, this study conducted a quantitative and visualized analysis of literature on septic shock in the past 30 years, revealing the core research, hotspot evolution and future trends in this field.

### 4.1 Current research status of septic shock

In the past three decades, the annual publication on septic shock has increased from 49 in 1996 to 297 in 2023, showing an upsurge year by year, particularly in the recent 3 years (Figure 2). This phenomenon showed that septic shock remained a major problem for health systems. Meanwhile, the medical community had an improved understanding of septic shock in the means of early identification, prevention and precise treatment. According to our analysis, the top 10 contributive countries (Table 1) were mostly developed countries such as Europe, the United States, and the Netherlands, among which the United States was the most productive country with various foreign cooperation. Two of the top 10 institutions in terms of publication volume (University of Pittsburgh and Harvard Medical School) were from the United States. The University of Pittsburgh showed strong cohesion in foreign cooperation and occupied a central position in the collaborative network. However, it had less cooperation with other top 10 institutions (University of Toronto, Columbia University, University of Amsterdam, etc.), indicating that a closer network had not yet been formed. China ranked fifth in the publication volume, and the top three productive institutions (Capital Medical University, Central South University, and Chinese Academy of Medical Sciences) were not listed in the top 10 of the world. These three institutions were relatively isolated in the network with little cross-national or cross-institutional collaboration. The high output of developed countries reflected their early start in research, as well as supportive scientific research foundation, national conditions and policy. There was still a large gap between China and other productive countries, requiring further improvement of the quality and academic influence of research literature.

The citation count reflects the influence of the author, and researchers can find cooperation by establishing a knowledge map of the co-author network. Among the co-cited authors in the field of septic shock (Figure 5A), scholar Vincent JL occupied a central position. In 2015, he led The Lancet Infectious Diseases Commission to summarize the pathogenesis, epidemiology, early identification and management of septic shock. He also pointed out that experimental studies in the future will continue to identify drug targets, while modify clinical trial design to benefit the patients from every clinical intervention (7, 16). In 2016, he, as the leader of a research team composed of the Society of Critical Care Medicine and the European Society of Intensive Care Medicine, updated the international consensus definition of sepsis and septic shock (Sepsis-3), and re-examined the clinical standards of sepsis/septic shock. As the study of sepsis becomes in-depth, its definition was no longer limited to a disease that originally required bacterial culture for diagnosis or was limited to inflammatory reactions caused by toxic substances of microorganisms. According to Sepsis-3, sepsis is defined as life-threatening organ dysfunction caused by a dysregulated host response to infection (11). This definition highlights and focuses on the dysregulated response to infection and organ dysfunction, which means sepsis is not limited to the potential danger of the infection itself, but also focuses on the complex pathophysiological reactions during infection. Septic shock is a subset of sepsis in which underlying circulatory and cellular/metabolic abnormalities are profound enough to substantially increase mortality, with characteristics such as complex disease, rapid changes, and a high mortality (11, 20). Notably, the Sepsis 3.0 incorporates the Sequential Organ Failure Assessment (SOFA) score and uses six criteria to reflect the function of organ systems (respiratory, coagulatory, liver, cardiovascular, renal, and neurologic). It serves as a bedside assessment tool for rapidly identifying patients with suspected exacerbations of infection who are at risk of poor prognosis, and covers the systems required for systemic inflammation or basic life maintenance (11). This update reflects the increasing understanding of the pathophysiology of sepsis and septic shock, as well as the need for more precise and clinically relevant diagnostic and treatment standards. The articles mentioned above were published in *Lancet Infectious Diseases* and *JAMA* (JCI Q1), respectively. The distribution map of journals identified the most published journals. As shown in Figure 4, *CRITICAL CARE MEDICINE* (JCI Q1) was the most cited journal, with 3,664 citations, making it the most prolific and influential journal in this field.

### 4.2 Hotspots in septic shock frontier

Through high-frequency keyword node analysis, time zone analysis, and citation burst analysis, we depicted the main research directions and trends in septic shock. Combining high-frequency keywords (Table 2) and spatiotemporal evolution trends (Figure 6C), the early research stage of septic shock focused on epidemiology, risk factors, disease management, and prognosis. As time progressed, research focuses shifted toward the multiple levels of pathogenesis (animals, cells, and molecules), as well as the novel drugs, early identification and diagnosis, and complications prevention. Among them, the identification and prevention of multiple organ dysfunctions such as “acute kidney injury,” “ARDS,” “myocardial injury,” and “DIC” have been active areas of exploration in this field (23). Studies have shown (24, 25) that the mechanisms of multi-organ dysfunction caused by septic shock have only been partially elucidated. The mechanism includes hypotension, reduced deformability of red blood cells, and microvascular thrombosis, which leads to reduced oxygen delivery and impaired tissue oxygenation. In addition, inflammation-induced endothelial dysfunction, accompanied by cell death and loss of barrier integrity, leads to subcutaneous and body cavity edema. Mitochondrial damage caused by oxidative stress can also impair the utilization of oxygen, thereby activating neutrophils and causing tissue damage. It is worth noting that genetic factors may also affect the susceptibility and outcome of sepsis among different populations. Genetic variations involved in immune response, such as genes encoding cytokines, toll-like receptors, and coagulation factors, have been shown to be associated with the risk and severity of septic shock (20, 26, 27). However, the role of genetic factors in septic shock remains complex and has not been fully understood, which is related to the individual heterogeneity, such as the presence of multiple pathogens, potential infection sites, and organ dysfunction (28). Further research is needed to clarify the interaction between genetic factors, environmental influences, and other risk factors.

The keyword burst diagram shows (Figure 7) that researchers have been committed to treatment of septic shock, and adjunctive drugs such as corticosteroids and immunomodulators have been extensively studied (29). At the same time, the use of advanced, external supportive care techniques such as extracorporeal membrane oxygenation and continuous renal replacement therapy has increased the treatment options for patients in developed countries. Unfortunately, despite decades of research, little progress has been made in the novel therapeutic agents for sepsis (30). The biological characteristics identified in septic shock have not yet been translated into effective new therapies. Highly specific drugs such as antithrombin, activated protein C, and anti-cytokines have not shown significant effects (31–33). As important components in the pathophysiology of septic shock, new therapeutic targets, including the endothelium and the microbiome, have been the focus of researches [173]. Glucocorticoids, an immunomodulatory drug used widely, have received the most attention as an adjunctive treatment for sepsis to regulate inflammatory responses and improve prognosis, but their therapeutic effects have also been controversial (34). Sepsis-3 recommends the use of low-dose corticosteroids based on the fact that patients with septic shock may still be hemodynamically unstable even after receiving evidence-based treatment (adequate fluid resuscitation and vasopressor) (35, 36). However, Sepsis-3 also points out the possible negative consequences, including hyperglycemia, gastrointestinal bleeding, and secondary infection (37).

And the only immunomodulatory therapy currently advocated is a short course of hydrocortisone for patients with refractory septic shock, but related clinical trials are still ongoing (38). Despite this, its effectiveness remains uncertainty and it is generally not recommended for use in clinical settings (39). At the same time, the specificity of the septic population, the complexity of the immune response, and the precise timing of intervention are also the reasons that hinder the development of effective immunomodulatory technologies (40). In addition, demographic factors such as age play an important role in the global burden of sepsis. Old age itself is a risk factor for predisposition to severe sepsis. Elder patients with septic shock often have serious comorbidities, which undoubtedly aggravate the host response and increase the risk of acute multi-organ dysfunction (8). Despite the use of antibiotics, fluid resuscitation, and intensive life support, death is still inevitable, which leads to a substantial increase in morbidity and mortality in the elderly individuals (23, 41). This is one of the reasons why the burden of septic shock is increasing due to the aging population in many countries, especially high-income countries (42). Given this, subsequent research should focus on identifying innovative treatment targets and strategies. The pathogenesis of complications, novel biomarkers, and the timing of alternative treatment will be the future hotspot and direction. In conjunction with precision medicine, future studies should include better preclinical models, more targeted drug development, and clinical trials with better patient selection, drug delivery, and outcome measurement (5).

Chinese scholars have also conducted a lot in septic shock, and the publication volume ranks fifth in the world, but the scale remains small. The most active domestic research institutions (Capital Medical University, Central South University, and Chinese Academy of Medical Sciences, etc.) are not listed in the world’s top 10, and lack cooperation with others. Therefore, it is imperative for Chinese scholars to accelerate research in related areas and to strengthen collaboration between countries by combining the current hotspots and evolution trends in septic shock.

## 5 Restrictions

It is important to acknowledge that our investigation has limitations. First, due to the limitations of the software and research methodology, we only selected literature from the WoSCC database. Most other databases, such as PubMed, Embase, and Scopus, do not have comprehensive information (full text and citation records), which was why we chose the WoSCC database. As a result, this may neglect the contribution of literature from other databases in this study field. In addition, the inclusion criteria were limited to English literature, and literature in other languages in the WoSCC database was not included. Although the subject searching included title, abstract, author keywords and Keywords Plus, it was still possible that relevant literature was not included in the statistics. Second, we only retrieved literature according to the inclusion and exclusion criteria. Consequently, our results primarily reflect the quantity, not the quality, of published studies, which may cause bias. Third, some high-quality publications may be overlooked in the literature analysis process due to their recent publication time and low number of citations. Fourth, since the database is updated dynamically, delays happen when performing bibliometric analysis. However, this does not significantly impact the general trends in this research area, and we believe that the conclusions drawn from our results cover the research hotspots and frontiers of septic shock, which offer valuable perspectives for future investigations.

## 6 Conclusion

Sepsis and septic shock are leading causes of death worldwide, and has become a major contributor to the global health burden. In this context, our study presents a comprehensive summary and analysis of the development trends and hot spots in septic shock. We retrieved septic shock-related literature in the past 30 years from the WoSCC database, and visualized the results by CiteSpace, VOSviewer and Pathfinder. Research on septic shock has rapidly progressed in the past decade. The pathogenesis of septic shock, novel biomarkers and alternative treatments are promising research areas. In addition, the rehabilitation trajectory of patients with septic shock has gained increasing attention and is expected to become a future research hotspot. Overall, this work may successfully give a research trajectory.

## Data Availability

The original contributions presented in the study are included in the article/supplementary material, further inquiries can be directed to the corresponding author.
